# Regression models and random effects

**DOI:** 10.1590/0100-6991e-20213210

**Published:** 2021-12-01

**Authors:** ÁLIDA ROSÁRIA SILVA FERREIRA

**Affiliations:** 1 - Universidade Federal De Minas Gerais, Estatística/Demografia/Nutrição - Belo Horizonte - MG - Brasil

When we think about carrying out any research, the most common questions are: over time, did the expected improvements happen? When comparing groups, does one stand out more than the other? What variables influence the behavior of another?

In this text, I will briefly talk about regression analysis. A statistical tool that aims to analyze the influence of one set of variables (named independent variables) over another (named dependent variable).

The biggest mistake made by anyone trying to use this tool is to blindly believe in mathematics without questioning the logic of what they are trying to do. For many years, while teaching undergraduate courses, I conducted the following experiment among my students: I asked them to count how many steps they walked since waking up until leaving home for college. I also asked them to count the number of yawns they gave throughout that time.

With all this information I tried to draw a relationship that would explain the number of yawns based on the number of steps. Most of the time, the relationship between these variables was significant. However, what does this tell us? Is there really a logical relationship between these two variables? Or was it just a fortunate mathematical coincidence? In this case, I tend to say that I was lucky or that the exposed time factor increased the chance of yawning. Anyway, there are countless cases that I have read in current published data that are 100% anchored in mathematics and do not discuss the clinical impact.

In regards with this topic, I will suggest a book that discusses exactly the performance of statistics, often disastrous in real cases: “Math on Trial: How Numbers Get Used and Abused in the Courtroom”. The readers will understand how numbers can build or destroy narratives.

The purpose of statistics is to be one of the pillars of a structure and not the only pillar. In times when confirmation bias is present in so many studies, it is important to be critical about what we read and produce.
